# Withaferin A Protects against Primary and Recurrent Tuberculosis by Modulating Mycobacterium-Specific Host Immune Responses

**DOI:** 10.1128/spectrum.00583-23

**Published:** 2023-03-14

**Authors:** Anjna Kumari, Isha Pahuja, Kriti Negi, Antara Ghoshal, Suparba Mukopadhyay, Meetu Agarwal, Babu Mathew, Jaswinder Singh Maras, Shivam Chaturvedi, Ashima Bhaskar, Ved Prakash Dwivedi

**Affiliations:** a Immunobiology Group, International Centre for Genetic Engineering and Biotechnology, New Delhi, India; b Department of Molecular Medicine, Jamia Hamdard University, New Delhi, India; c Department of Molecular and Cellular Medicine, Institute of Liver and Biliary Sciences, New Delhi, India; Griffith University

**Keywords:** withaferin A, immunotherapy, tuberculosis, memory T cells, disease relapse, *Mycobacterium tuberculosis*, tuberculosis vaccines

## Abstract

The fate of Mycobacterium tuberculosis infection is governed by immune signaling pathways that can either eliminate the pathogen or result in tuberculosis (TB). Anti-TB therapy (ATT) is extensive and is efficacious only against active, drug-sensitive strains of M. tuberculosis. Due to severe side effects, ATT often causes impairment of host immunity, making it imperative to use novel immunotherapeutics for better clinical outcomes. In this study, we have explored the immunomodulatory potential of withaferin A (WA) as an immunotherapeutic against TB. Here, we demonstrate that WA can constrain intracellular drug-sensitive and -resistant strains of M. tuberculosis by augmenting host immune responses. We also established the potential of WA treatment in conjunction with isoniazid. We show that WA directs the host macrophages toward defensive M1 polarization and enhances T_H_1 and T_H_17 immune responses against M. tuberculosis infection. The reduced bacterial burden upon T cell adoptive transfer further corroborated the augmented T cell responses. Interestingly, WA stimulated the generation of T cell memory populations by instigating STAT signaling, thereby reducing the rate of TB recurrence due to reactivation and reinfection. We substantiate the prospects of WA as a potent adjunct immunomodulator that enriches protective memory cells by prompting STAT signaling and improves host defense against M. tuberculosis.

**IMPORTANCE** Despite being extensive, conventional antituberculosis therapy (ATT) is barely proficient in providing sterile immunity to tuberculosis (TB). Failure to constrain the escalating global TB burden due to the emergence of drug-resistant bacterial strains and immune dampening effects of ATT necessitates adjunct immunotherapeutics for better clinical outcomes. We evaluated the prospects of withaferin A (WA), an active constituent of Withania somnifera, as an adjunct immunomodulator against diverse M. tuberculosis strains. WA efficiently restricts the progression of TB by stimulating antimycobacterial host responses, protective immune signaling, and activation of diverse immune cell populations. Protective effects of WA can be attributed to the enrichment of memory T cells by induction of STAT signaling, thereby enhancing resistance to reinfections and reactivation of disease. We ascertained the immunotherapeutic potential of WA in boosting host immune responses against M. tuberculosis.

## INTRODUCTION

The search to elevate conventional antituberculosis therapy (ATT) has directed researchers to explore progressive approaches to halt the eternal tuberculosis (TB) pandemic. Mycobacterium tuberculosis was first identified as the causative agent of TB in 1882 by Robert Koch. We have scientifically come a long way since then, but despite accessibility of the standard antimycobacterial antibiotics and prophylactic vaccine, about one-fourth of the world population is asymptomatically suffering from latent TB. TB is preventable and even with the availability of treatment, 10 million people are diseased with TB annually (1).

Standard ATT can proficiently exterminate active, drug-sensitive strains of M. tuberculosis in 6 to 8 months. However, failure to complete the prolonged directly observed treatment short course (DOTS) frequently brings about the emergence of drug-resistant (DR) strains. Unresponsiveness to established anti-TB drugs in DR-TB patients endures as a deadly safety hazard (1–3). Moreover, DOTS therapy may result in impairment of host immune responses, thereby heightening the risk of reactivation and reinfection with M. tuberculosis (4–6). The major concern regarding ATT is that it does not include an immunomodulator, which is recommended by WHO expert consultation to constrain adverse effects on the host (7). It is the need of the hour to reinforce the standard anti-TB therapy with adjunct immunomodulatory therapeutics to end the TB epidemic by 2030 (1).

The majority of individuals can restrain M. tuberculosis infections owing to immune responses capable of constraining infection (8). It is well established that dynamic immune responses of diverse T cell subsets contribute to governing the consequences of M. tuberculosis infections (9). Macrophages elicit chemokines and cytokines in response to M. tuberculosis infection, and triggered proinflammatory responses direct migration of other subsets of immunity at the site of infection. Induction of T_H_2 cells and regulatory T cells subsequent to M. tuberculosis infection intensifies disease progression by impeding protective T_H_1 responses (10–12). Induction of proinflammatory cytokines such as gamma interferon (IFN-γ) and interleukin 17 (IL-17) is required for differentiation of naive CD4^+^ T cells into T_H_1 and T_H_17 cells, which stimulate a plethora of host-protective responses and play important roles in eliciting recall immune responses against reinfections (13). Nevertheless, immunoregulatory cytokines and regulatory T cells (Tregs) also play a critical role in controlling excessive inflammation (14). However, this defensive immune response contracts subsequent to bacterial clearance, and subsets of memory T cells are involved in long-term immunity (15). T effector memory (T_EM_) cells protect against acute M. tuberculosis infections by eliciting T_H_1-type cytokines, and T central memory (T_CM_) cells maintain long-term memory responses (16, 17). Additionally, T_CM_ cells can generate T_EM_ cells in the course of ongoing infection and drive cell-mediated host immune responses to exterminate bacteria (18). Tissue-resident memory T (T_RM_) cells are actively engaged in stimulating protective immune responses at specific sites of infection, such as lung and spleen, and are associated with favorable clinical outcomes (19). Finally, T stem cell memory (T_SCM_) is a rare subset of memory T cells that can self-renew and establish the absolute continuum of memory and effector T cells.

Phytochemicals characterized from plant extracts are beneficial owing to minimal side effects, ease of availability, and a broad spectrum of antiviral and antibacterial properties. Across the world, researchers are identifying and demonstrating the antitubercular potential of many compounds and inhibitors independently and in combination with existing drugs (20). Withania somnifera, commonly known as ashwagandha, is a medicinal plant that is widely employed to relieve stress and revitalize the host (21). In phase 1 clinical trials, W. somnifera was found to be well tolerated, with no significant adverse effects or hepatotoxicity (22). Among diverse withanolides isolated from W. somnifera, withaferin A (WA) is the most prominent bioactive steroidal lactone, known for antioxidative, anti-lipid peroxidative, and detoxifying properties (23, 24), and research groups have evaluated the broad-spectrum immunomodulatory potential of WA against microbial infections (25). On the other hand, corticosteroids are generally immunosuppressive in nature and have various adverse effects during long-term uses (26).

In this study, we set out to identify the antimycobacterial potential of WA against a pathogenic strain of M. tuberculosis, H37Rv, and drug-resistant clinical isolates, the multidrug-resistant (MDR) isolate JAL-2261 and the extensively drug-resistant (XDR) isolate MYC-431, by systematically performing *in vitro*, *ex vivo*, and *in vivo* experiments. With no direct antimycobacterial activity, WA significantly induced the intracellular killing of M. tuberculosis by modulating vital signaling cascades to induce reactive oxygen species (ROS) generation, proinflammatory cytokine secretion, and enhanced costimulatory molecule expression. Furthermore, WA treatment augmented the activation of CD4^+^ and CD8^+^ T cells *ex vivo*. Collective immunomodulatory impact of WA on macrophages and T cells was further validated via coculture experiments, with significant reduction in infection and bacterial burden upon WA treatment. WA also demonstrated strong immunomodulatory properties in M. tuberculosis-infected mice by enhancing the host protective innate and adaptive immune responses. Further, WA significantly reduced bacterial load in a mouse model of TB either alone or in combination with the first-line anti-TB drug isoniazid (INH) and significantly improved the immune dampening effects of INH. Enrichment of M. tuberculosis-specific T cell responses was further validated by reduction in bacterial burden upon adoptive transfer of CD3^+^ T cells in Rag1^−/−^ mice. Interestingly, WA treatment enriched memory T cell populations, namely, T_CM_, T_EM_, T_RM_, and T_SCM_ populations. WA-induced host-protective responses were further validated via *ex vivo* experiments which linked stimulation of STAT3 and STAT4 signaling pathways with enhanced CD4^+^ and CD8^+^ T cell responses. Furthermore, superior induction of memory responses led to a significant reduction in TB relapse and TB reinfection with concurrent enrichment of CD8^+^ T_CM_ cells, thereby corroborating WA as a potential adjunct immunotherapeutic against TB.

## RESULTS

### WA enhances host defenses against M. tuberculosis infection.

**FIG 1:**
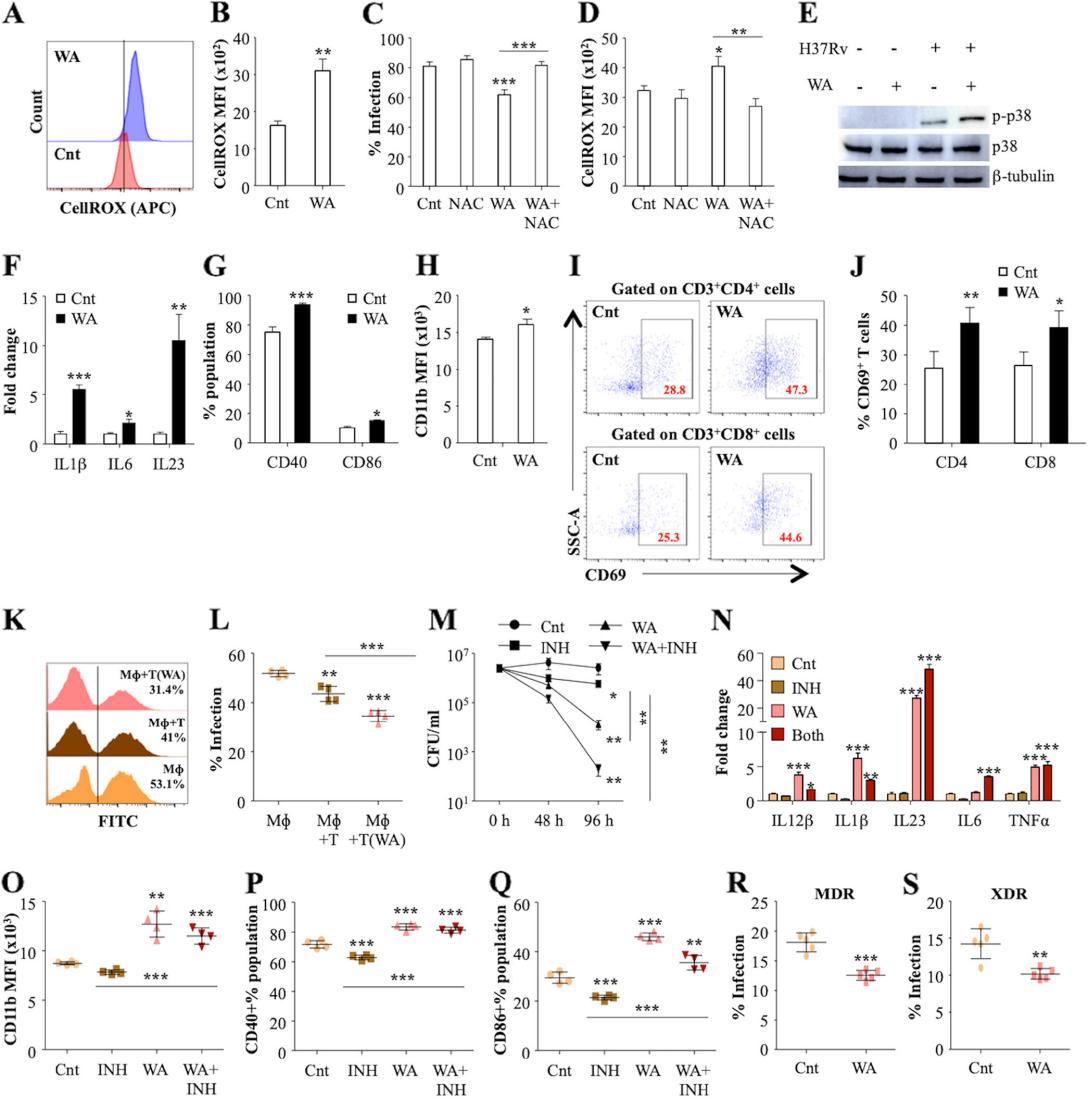
WA induces resistance against TB. Mouse peritoneal macrophages were infected with M. tuberculosis H37Rv at an MOI of 1:10, followed by treatment with WA (0.5 μg/mL). At 48 h postinfection (p.i.), cells were processed for ROS analysis. (A and B) Representative histograms (A) and bar graph (B) showing CellROX mean fluorescence intensity (MFI) in the cells with and without WA treatment. Mouse peritoneal macrophages pretreated with 10 mM NAC were infected with green fluorescent protein (GFP)-expressing M. tuberculosis H37Rv at an MOI of 1:1, followed by treatment with WA. (C) Percentage of M. tuberculosis-infected macrophages at 48 h pi. (D) Corresponding levels of ROS in the infected macrophages. (E) Immunoblots depicting the phosphorylation status of p38 in uninfected and infected mouse peritoneal macrophages with or without WA treatment. (F) Proinflammatory cytokine expression levels in the infected macrophages with or without WA treatment. (G) Percentages of infected macrophages expressing CD40 and CD86 with or without WA treatment. (H) CD11b expression on infected macrophages with and without WA treatment. (I and J) Splenocytes isolated from M. tuberculosis-infected mice were *ex vivo* stimulated with M. tuberculosis complete soluble antigen (CSA) and treated with WA (0.1 μg/mL) for 48 h, followed by surface staining with anti-CD3, -CD4, -CD8, and -CD69. (I) Representative dot plots showing percentage activation (CD69 expression) on CD4^+^ T cells and CD8^+^ T cells in the splenocytes with and without WA treatment. (J) Quantification of the CD69 expression on CD4^+^ and CD8^+^ T cells with and without WA treatment. (K and L) Mouse peritoneal macrophages infected with GFP-expressing H37Rv (Rv-GFP) at an MOI of 1:1 were cocultured with WA-primed T cells for 48 h, followed by flow cytometry. (K) Histogram representing the GFP fluorescence in different experimental conditions. (L) Percentage infection in macrophages (Mɸ) alone, Mɸ plus T cells, and Mɸ plus T cells pretreated with WA. (M to Q) Mouse peritoneal macrophages were infected with M. tuberculosis H37Rv at an MOI of 1:10, followed by treatment with WA (0.5 μg/mL) and INH (0.5 μg/mL). (M) CFU enumeration in infected macrophages either untreated or treated with INH, WA, and INH plus WA at different time points. (N) Proinflammatory cytokine expression levels in the infected macrophages either untreated or treated with INH, WA, and INH plus WA. (O) Graph showing CD11b expression on infected macrophages either untreated or treated with INH, WA, and INH plus WA. (P and Q) Percentages of CD40 (P) and CD86 (Q) expression in infected macrophages either untreated or treated with INH, WA, and INH plus WA. (R and S) Percent infection in MDR (R) and XDR (S) strain-infected macrophages either untreated or treated with WA. The data are representative of three independent experiments.

Fragmentary information regarding the therapeutic potential of WA against numerous diseases has been presented by several studies (27, 28). However, even with distinct evidence of clinical recovery in TB patients (22), the efficacy of WA to treat TB remains to be confirmed. We assessed the direct antibacterial activity of WA against a virulent strain of M. tuberculosis (H37Rv) via alamarBlue assay and observed that WA did not exhibit a direct antimycobacterial activity at the tested concentrations (see Fig. S1A and B in the supplemental material). Prior to *ex vivo* infection experiments, we ascertained a safe dosage of WA (0.5 μg/mL) through staining of murine macrophages treated with range of WA concentrations with propidium iodide (PI) dye (Fig. S1C). Subsequently, the immunomodulatory effects of WA treatment were evaluated in mouse peritoneal macrophages infected with M. tuberculosis H37Rv at a multiplicity of infection (MOI) of 1:1. In response to M. tuberculosis internalization, reactive oxygen species (ROS) are released by infected macrophages as an innate defense mechanism to kill the intracellular bacilli (9). In M. tuberculosis-infected peritoneal macrophages, WA treatment enhanced ROS generation in response to M. tuberculosis infection (Fig. 1A and B). The impact of intensified ROS generation on the survival of intracellular M. tuberculosis was further evaluated in murine macrophages. For this, peritoneal macrophages pretreated with 10 mM N-acetyl cysteine were infected with H37Rv-GFP at an MOI of 1:1 and were treated with WA for 48 h, followed by flow cytometry. WA treatment significantly reduced the percentage of M. tuberculosis infection in macrophages, with an equivalent increase in the intracellular ROS, while NAC treatment diminished the antimycobacterial activity of WA by reducing the levels of ROS in the macrophages (Fig. 1C and D). In response to M. tuberculosis infection, macrophages activate several immune signaling pathways to secrete proinflammatory cytokines to fortify host defenses (8). Interestingly, in M. tuberculosis-infected macrophages, WA treatment activated the p38 mitogen-activated protein kinase (MAPK) signaling pathway (Fig. 1E), which is linked with activation of proinflammatory responses to counteract M. tuberculosis infection (29). Correspondingly, WA treatment significantly increased the expression of proinflammatory cytokines, such as IL-6, IL-1β, and IL-23, in M. tuberculosis-infected macrophages (Fig. 1F). IL-23 and IL-1β were recently shown to drive T_H_17 differentiation, critical for recall responses (30–32). Macrophages process bacilli and present antigens to T cells via upregulation of costimulatory molecules such as CD40, CD80, CD86, and major histocompatibility complex class II (MHC-II). We observed that WA treatment elevated the expression of costimulation markers on macrophages in response to M. tuberculosis infection (Fig. 1G), with an analogous increase in expression of CD11b (Fig. 1H). In the later phase of infection, acquired immunity against M. tuberculosis paradigmatically depends on specific T lymphocytes. The macrophages and T cells interact with each other to determine the outcome of the infection. Early T cell activation is critical for control of M. tuberculosis infection. Surprisingly, WA treatment significantly enhanced the percentage of activated CD4^+^ and CD8^+^ T cells (Fig. 1I and J). To examine the effect of WA on macrophages and T cells collectively, we replicated the *in vivo* conditions and performed coculture experiments. Splenocytes isolated from M. tuberculosis-infected mice were pretreated with WA prior to coculture with M. tuberculosis-infected peritoneal macrophages. WA pretreatment significantly enhanced the killing capacity of T cells, as evidenced by reduction in the bacterial infection in the macrophages cocultured with WA-primed T cells compared to other groups (Fig. 1K and L).

Moreover, to examine the efficacy of WA as an adjunct to anti-TB therapy, we performed experiments with the front-line drug INH alone and in combination with WA. In infected macrophages, WA treatment alone and together with INH significantly reduced the intracellular bacterial growth compared to those obtained with the control (Cnt) and INH alone (Fig. 1M). Furthermore, WA treatment alone or in conjunction with INH significantly induced the expression of protective cytokines (IL-12β, IL-1β, IL-23, IL-6, and tumor necrosis factor alpha [TNF-α]) in infected macrophages (Fig. 1N). Interestingly, INH-induced reduction in the expression of costimulatory molecules was abrogated by WA (Fig. 1O to Q). Lastly, we determined the antimycobacterial potential of WA for drug-resistant strains. Similar to the case with the drug-sensitive strain, WA treatment reduced the bacterial infection in macrophages infected with fluorescently stained MDR and XDR strains of M. tuberculosis (Fig. 1R and S). Overall, the *ex vivo* results imply that WA induces the activation of macrophages and prompts polarization toward the M1 phenotype upon M. tuberculosis infection. WA treatment also elevates the T cell responses, thereby increasing host resistance. Interestingly, WA showed a synergistic effect with INH, and the antimycobacterial efficacy of WA was not limited to the drug-sensitive strain, which demonstrates prospects of WA as an adjunct immunotherapeutic against TB.

### WA treatment restricts mycobacterial growth and detrimental pathology in M. tuberculosis-infected mice.

**FIG 2:**
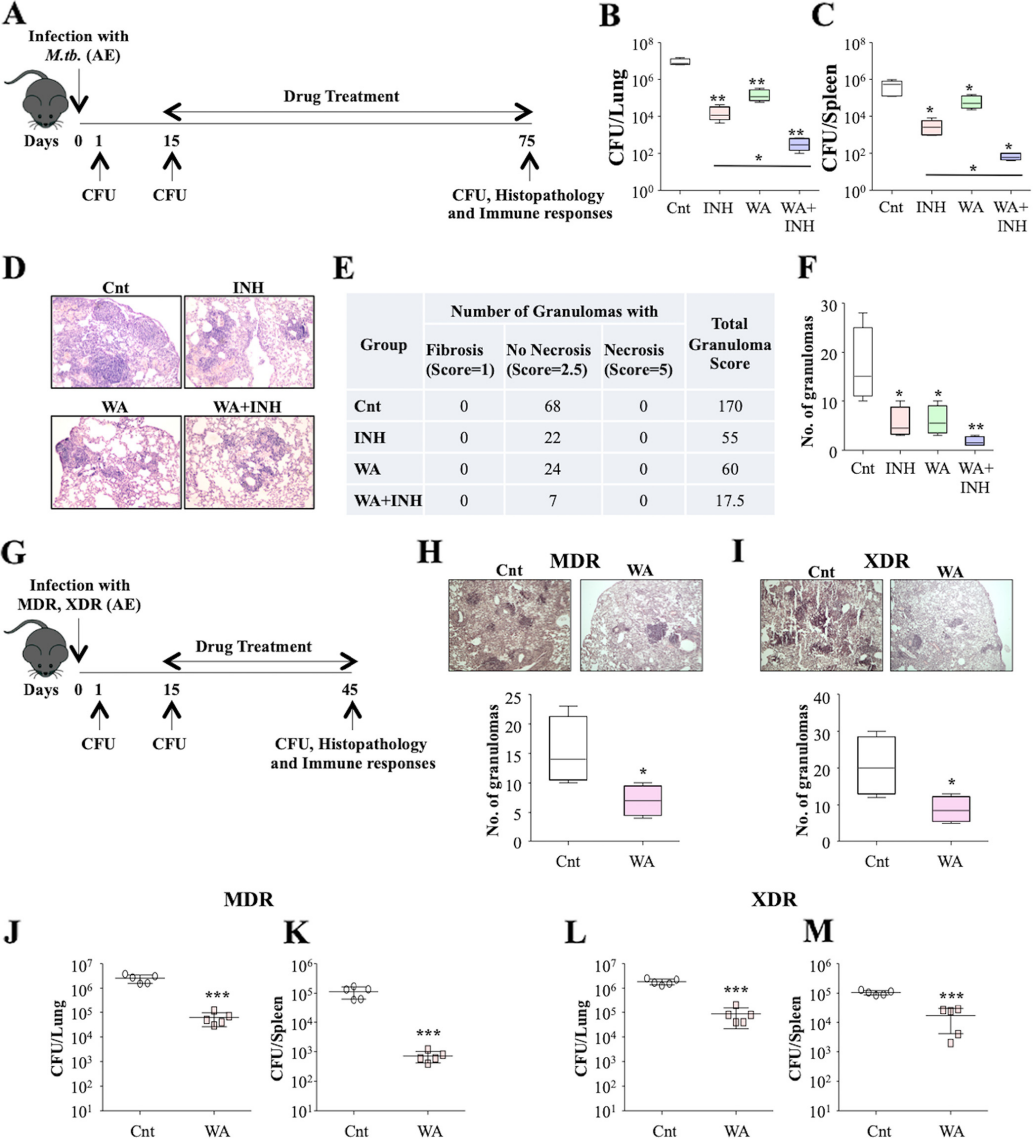
WA elevates host-protective effects in murine model of TB. (A) C57BL/6 mice were challenged with H37Rv (low dose; ~110 CFU/mice) via the aerosol route, and necropsy of these mice treated with WA and INH was done at 75 days p.i. to harvest the lungs and the spleen for CFU enumeration, histopathology, and immune profiling. (B and C) Determination of the bacterial burden in the lung (B) and spleen (C) homogenates of infected mice. (D) Representative histopathology images from the lungs harvested from infected mice. (E and F) Quantification of the lung granuloma (inflammatory lesions) in all experimental groups. Further WA treatment reduces the pathophysiology against drug-resistant clinical isolates of M. tuberculosis. (G) Mouse infection model representing the study design. (H and I) Histopathology images and quantification of the total number of granulomas in the lungs infected with MDR strain (H) and (I) XDR strain of M. tuberculosis. (J and K) Bacterial burden in the lung (J) and spleen (K) homogenates of MDR strain-infected mice. (L and M) Bacterial burden in the lungs (L) and spleens (M) of XDR strain-infected mice. Data are representative of two independent experiments with five mice per group.

To better comprehend *ex vivo* outcomes and corroborate the dramatic reduction in bacterial load upon WA treatment alone or in combination with INH, we performed an *in vivo* study in the murine model of TB. C57BL/6 mice were challenged with a low dose (~110 CFU) of the virulent strain M. tuberculosis H37Rv. Groups of mice were either kept untreated (Cnt) or were treated with WA (2 mg/kg of body weight) alone or with INH (100 mg/liter) for 60 days, followed by CFU enumeration, histopathology, and immunological analysis (Fig. 2A). WA treatment significantly lowered the bacillary loads in the lungs and the spleens of M. tuberculosis-infected mice compared to those of the Cnt group (Fig. 2B and C). Furthermore, a synergistic effect in the bacterial reduction was observed in the infected mice treated with WA and INH, indicating that WA cotreatment extensively enhanced the antitubercular potential of INH (Fig. 2B and C). Histopathological examination of the lungs revealed that WA treatment alone or in combination with INH noticeably reduced the necrotic lesions associated with granulomas in M. tuberculosis-infected mice (Fig. 2D to F). These results demonstrate that WA enhances host defense against drug-sensitive strain H37Rv.

It is extremely challenging to treat multidrug-resistant TB (MDR-TB) and extensively drug-resistant TB (XDR-TB) utilizing standard TB treatment. Therefore, MDR-TB and XDR-TB warrant innovative therapeutic strategies. Plant-based immunomodulatory compounds possess the capability to target drug-sensitive as well as drug-resistant strains of M. tuberculosis (33). Prospective *in vivo* outcomes against H37Rv prompted us to further assess the efficacy of WA treatment against drug-resistant clinical isolates of M. tuberculosis. To this end, C57BL/6 mice were infected with MDR or XDR strains of M. tuberculosis and after 30 days of treatment with WA were sacrificed for CFU and immunological analysis (Fig. 2G). Histopathological analysis of lungs isolated from both MDR and XDR strain-infected mice demonstrated reduction in detrimental pathology in the WA-treated group compared to the control (Fig. 2H and I). Similar to the case with the drug-sensitive strain H37Rv, remarkable reductions in bacterial burdens were observed in the lungs and the spleens of MDR (Fig. 2J and K) and XDR (Fig. 2L and M) strain-infected mice. Thus, WA strikingly reduced M. tuberculosis load and pathology associated with bacilli in both drug-sensitive and drug-resistant M. tuberculosis strain-infected mice, establishing the potential of WA as an adjunct to existing ATT.

### WA strengthens innate and adaptive immune responses against M. tuberculosis.

**FIG 3:**
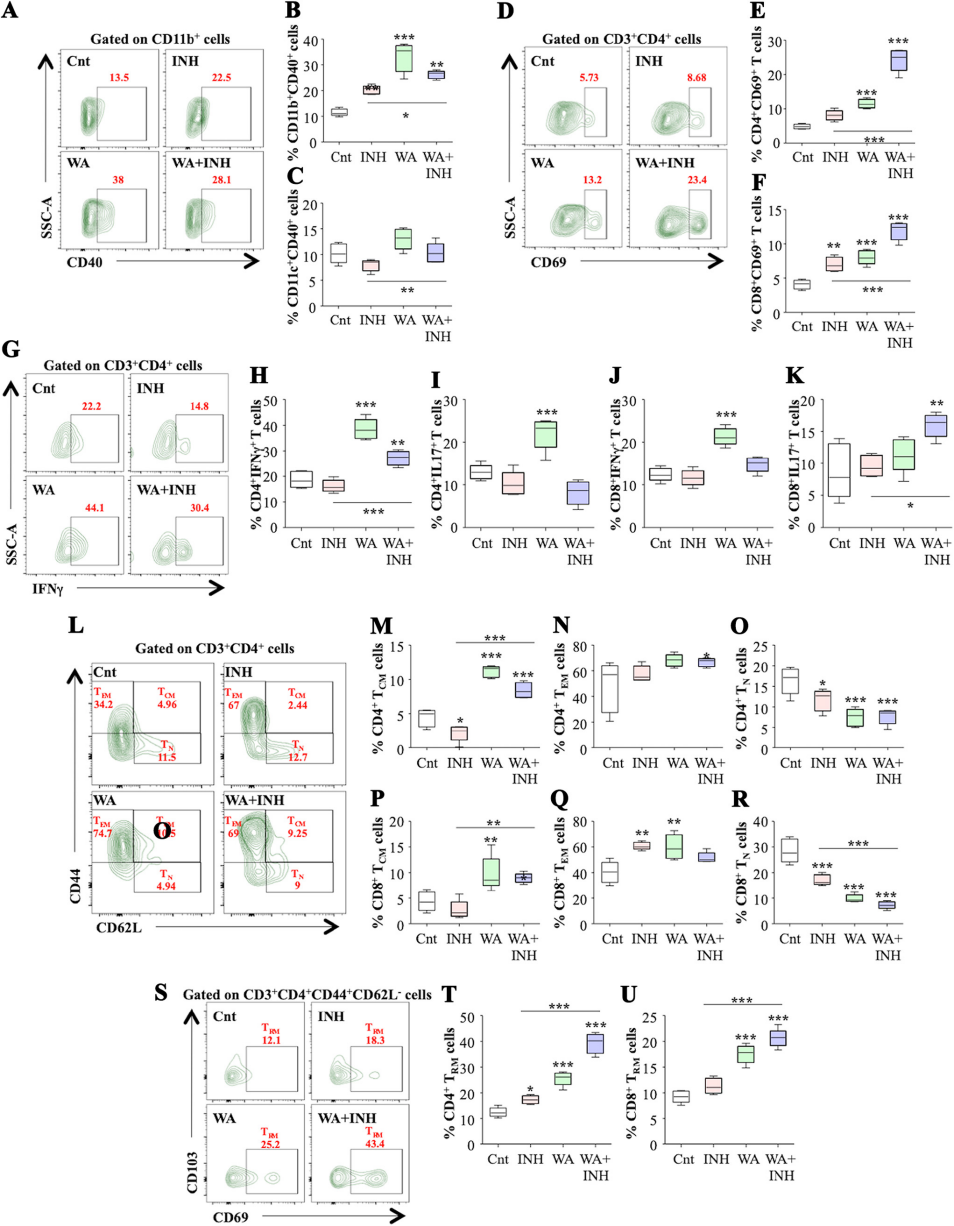
WA induces long-term protection against tuberculosis by enriching central memory T cells. *Ex vivo*-stimulated lung cells were stained with different antibodies directed against surface markers and intracellular cytokines followed by flow cytometry. (A to C) Representative contour plots (A) and quantification of CD11b^+^ CD40^+^ (B) and CD11c^+^CD40^+^ (C) cells in the lungs of infected mice. (D) Representative contour plots showing percent activation (CD69 expression) on CD4^+^ T cells in the infected lungs. (E and F) Quantification of the CD69 expression on CD4^+^ (E) and CD8^+^ (F) T cells. (G) Contour plots depicting CD3^+^ CD4^+^ IFN-γ^+^ cells in all experimental groups. (H and I) Percentages of CD4^+^ cells producing IFN-γ (H) and IL-17 (I) in the lungs of infected mice. (J and K) Percentages of IFN-γ-producing (J) and IL-17-producing (K) CD8^+^ T cells in the lungs of infected mice. (L) Contour plots demonstrating various memory T cell subsets under different experimental conditions. (M to O) Percentages of CD4^+^ CD62L^HI^ CD44^HI^ (T_CM_) cells (M), CD4^+^ CD62L^LO^ CD44 ^HI^ (T_EM_) cells (N), and CD4^+^ CD62L^HI^ CD44^LO^ (T_N_) cells (O) in the lungs of infected mice. (P to R) Bar graphs representing the percentage of CD8^+^ CD62L^HI^ CD44^HI^ (T_CM_) cells (P), CD8^+^ CD62L^HI^ CD44^LO^ (T_EM_) cells (Q), and CD8^+^ CD62L^HI^ CD44^LO^ (T_N_) cells (R) in the lungs of infected mice. (S) Representative contour plots of T_RM_ cells (CD3^+^ CD4^+^ CD44^+^ CD62L-CD69^+^ CD103^+^) in different experimental groups. (T and U) Percentages of CD4^+^ T_RM_ cells (T) and CD8^+^ T_RM_ cells (U) in the lungs of infected mice. Data are representative of two independent experiments with five mice per group.

For a deeper understanding of the immunomodulatory effects of WA, we profiled different innate and adaptive immune cell populations which drive host-protective responses against M. tuberculosis infection in the lungs and spleens of M. tuberculosis-infected mice. Figure S2 shows the gating strategy employed for immune profiling. Stimulation of innate immune cells triggers activation of antigen-specific adaptive immune responses (8). WA treatment significantly boosted the percentage of activated antigen-presenting cell (APC) populations, namely, macrophages (CD11b^+^ CD40^+^) and dendritic cells (CD11c^+^ CD40^+^), in the lungs (Fig. 3A to C) and the spleens (Fig. S3A and B) of infected mice. Corresponding to the enhanced population of activated innate immune cells, the stimulated adaptive immune cell population was also augmented in WA-treated infected mice. WA treatment significantly enhanced the percentage of CD4^+^ and CD8^+^ T cells expressing early activation marker CD69 in the lungs (Fig. 3D to F) and spleens (Fig. S3C and D) of infected mice. Interestingly, WA cotreatment with isoniazid showed the synergistic influence on the activation of innate and adaptive immune cells, as higher percentages of activated APCs and T cells were observed on cotreatment than for INH alone (Fig. 3A to F). Therefore, WA treatment alone or in combination with INH significantly enhanced the percentage of activated innate cell populations, which consequently promoted the expansion of activated T lymphocytes in response to M. tuberculosis infection. A central tenet of defense against M. tuberculosis is to aid the T_H_1/T_H_17 immune responses, leading to better control over disease progression. WA treatment significantly increased IFN-γ- and IL-17-producing T cell subsets in both the lungs (Fig. 3G to K) and spleens (Fig. S3E-3H) of infected mice. Hence, it can be inferred that WA treatment skews T cell responses toward T_H_1/T_H_17 and elevates protective proinflammatory responses. Further analysis revealed that WA treatment significantly upregulated CD4^+^ T cells expressing IL-2 and TNF-α in the spleens of infected mice, with no effect seen in the lungs (Fig. S3I and J). However, polyfunctional CD4^+^ T cells which expressed IFN-γ, IL-17, IL-2, and TNF-α were significantly higher in both the spleens and lungs of WA-treated infected mice than in the untreated control mice (Fig. S3K and L). Subsequently, we investigated the impact of WA treatment on immune cell populations in the MDR and XDR strain-infected mice. While there was no difference in the percentages of CD4^+^ and CD8^+^ T cells, WA treatment significantly enhanced the activated CD4^+^ and CD8^+^ T cell populations in the lungs of MDR and XDR strain-infected animals (Fig. S4A to D), with no significant difference in the spleen (Fig. S4E to H). Similar to the case with mice infected with H37Rv, WA treatment skewed the host immunity toward T_H_1/T_H_17 responses in the lungs of MDR and XDR strain-infected mice (Fig. S4I to L), while a modest effect was observed in the spleens (Fig. S4M to P). In summary, these observations reveal the immunomodulatory effects of WA during M. tuberculosis infection with drug-sensitive as well as drug-resistant strains.

Prolonged usage of numerous drugs in the course of DOTS can induce lethal hepatotoxicity, which is also associated with the inability to complete therapy and increases the risk of developing resistance to available drugs (34). Therefore, we assessed the influence of WA on subsiding DOTS-associated hepatotoxicity. Serum glutamic pyruvic transaminase (SGPT) and serum glutamic oxaloacetic transaminase (SGOT) levels were estimated in different groups of M. tuberculosis-infected mice. Consistent with previous studies, administration of INH plus rifampin (RIF) (DOTS) induced hepatotoxicity in M. tuberculosis-infected mice (Fig. S5). WA treatment mended DOTS-induced hepatotoxicity, which was evident with the reduction in serum SGPT and SGOT levels in the sera of mice treated with WA along with INH (Fig. S5). Thus, WA as an adjunct to DOTS may further improve therapeutic outcomes by easing DOTS-induced hepatotoxicity and impairment leading to withdrawal and rise of drug resistance.

### WA enriches M. tuberculosis-specific memory T cell responses.

It is well established that memory T cells play a vital role in eliciting antigen-specific recall responses. Two chief memory T cell subsets, central memory T (T_CM_) cells and effector memory T (T_EM_) cells, have distinct characteristics and functions. T_CM_ cells can efficaciously proliferate after antigen stimulation, whereas the T_EM_ population exhibits intensified effector functions with restricted proliferative capacity. Moreover, T_CM_ cells can give rise to T_EM_ cells, which drive bacterial clearance. Hence, augmenting the pool of T_CM_ cells may be an effective strategy to develop long-lasting immunity against M. tuberculosis. Therefore, we investigated the impact of WA treatment on different T cell memory subsets within the CD4^+^ and CD8^+^ T cell populations (Fig. 3L). We observed a significant increase in the percentage of CD4^+^ T_CM_ cells (Fig. 3M), with no effect on CD4^+^ T_EM_ population (Fig. 3N), in the lungs of infected mice. There was a concomitant decrease in the percentage of naive CD4^+^ T (T_N_) cells (Fig. 3O), implying WA-induced differentiation of naive T cells into different memory T cell subsets. A similar trend was observed in CD8^+^ T cells (Fig. 3P to R). Even the spleens of WA-treated infected mice displayed a significant increase in the central memory T cell subsets (Fig. S3I and J). Moreover, WA coadministration significantly enriched the pool of T_CM_ cells in the lungs (Fig. 3M and P) and spleens (Fig. S3I and J) of INH-treated mice. We further investigated the influence of WA on resident memory T (T_RM_) cells, which are known to reside at specific tissues and progress from disseminating T_EM_ cells. WA treatment considerably enhanced the percentages of CD4^+^ and CD8^+^ T_RM_ cells in the lungs (Fig. 3S to U) as well as in the spleens (Fig. S3K and L) of infected mice. Together, these results suggest that WA treatment may strengthen the recall response by expanding T_CM_ and T_RM_ cell populations in infected mice.

### Antimycobacterial potential of WA is associated with protective T cell responses.

**FIG 4:**
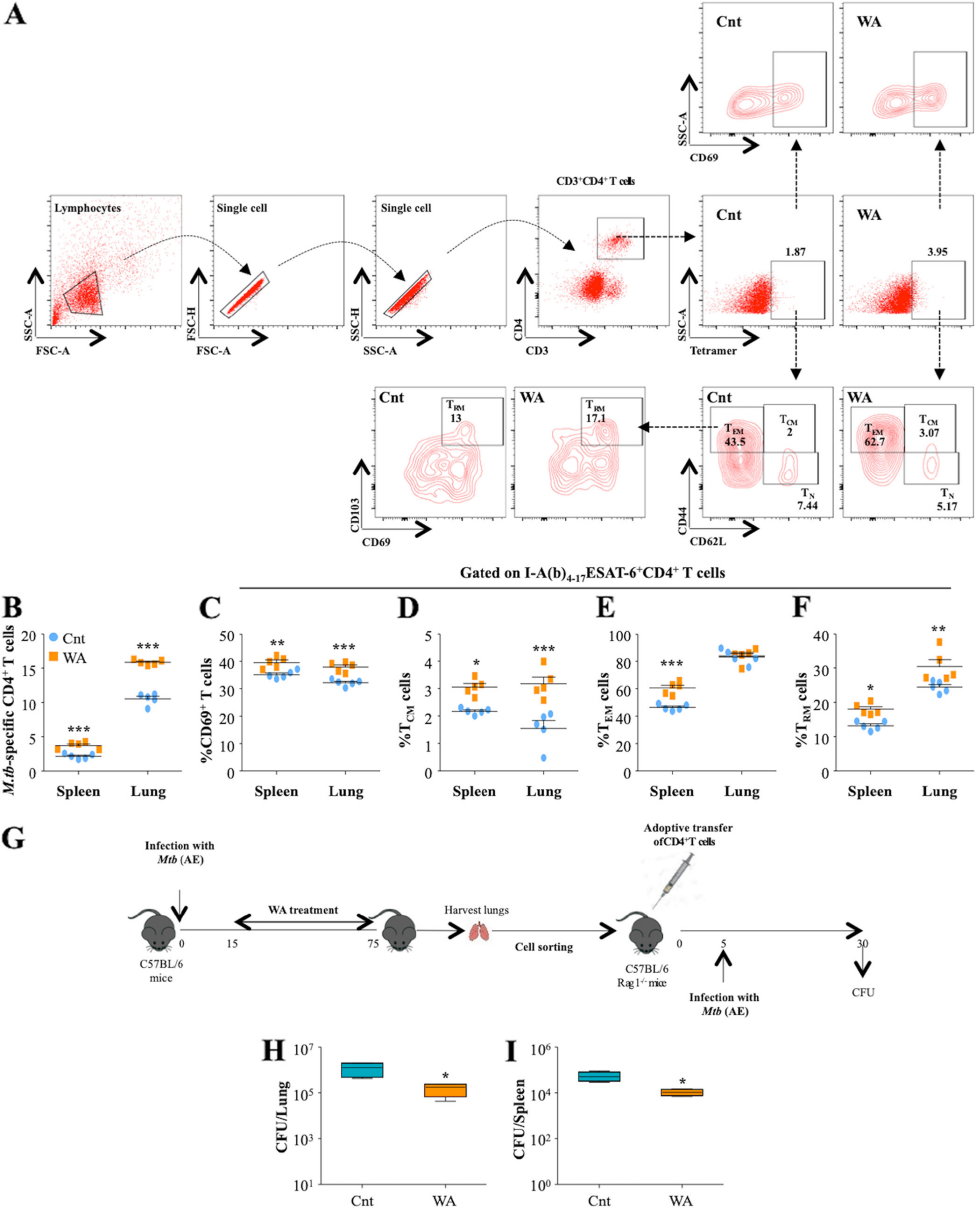
WA provides T cell-mediated protection against M. tuberculosis*. Ex vivo*-stimulated lung and spleen cells were stained with different antibodies directed against surface markers and I-A(b)_4-17_ESAT6 MHC-II tetramer, followed by flow cytometry. (A and B) Gating strategy (A) and representative dot plots indicating the quantification (B) of I-A(b)_4-17_ESAT6 MHC-II-specific CD4^+^ T cells in the spleens and the lungs of infected mice. (C to F) Proportions of CD69^+^ (C), T_CM_ (D), T_EM_ (E), and T_RM_ (F) cells among I-A(b)_4-17_ESAT6 MHC-II-specific CD4^+^ T cells. (G) Experimental layout of the study. C57BL/6 mice were infected with H37Rv, followed by WA treatment for 60 days. CD4^+^ T cells were sorted from the lungs of infected mice and adoptively transferred into Rag1^−/−^ mice, followed by infection with H37Rv. At 25 days after infection, CFU were estimated from the lung (H) and spleen (I) homogenates of the different groups. Data are representative of two independent experiments with five mice per group.

Our data strongly suggested that WA treatment modulates host immune responses to confer protection against TB. In order to examine if these responses were M. tuberculosis specific, we used an I-A(b)_4-17_ESAT-6 MHC-II tetramer to analyze the M. tuberculosis-specific CD4^+^ T cell responses in the lungs and spleens of infected mice upon WA treatment (Fig. 4A). To our satisfaction, WA treatment significantly increased the percentage of ESAT-6-specific CD4^+^ T cells in the lungs and spleens of infected animals (Fig. 4B). Further immune phenotyping revealed significant increases in the activation and memory responses upon WA treatment (Fig. 4C to F). To further corroborate that WA-induced protective immunity was a consequence of M. tuberculosis-specific CD4^+^ T lymphocytes, we performed an adoptive-transfer study. CD4^+^ T cells were sorted from the lungs of infected and WA-treated mice and were intravenously transferred into Rag1^−/−^ mice, which lack mature B and T lymphocytes (35), followed by low-dose M. tuberculosis infection; CFU were enumerated 25 days postinfection (p.i.) (Fig. 4G). We found significant decreases in the bacterial loads in the lungs and the spleens of mice that received T cells from WA-treated animals compared to the control group (Fig. 4H and I), substantiating that WA-induced enhancement of antimycobacterial responses is the result of M. tuberculosis-specific T cell immune responses.

### WA treatment promotes M. tuberculosis-specific memory responses by modulating STAT3 and STAT4 signaling pathways.

**FIG 5:**
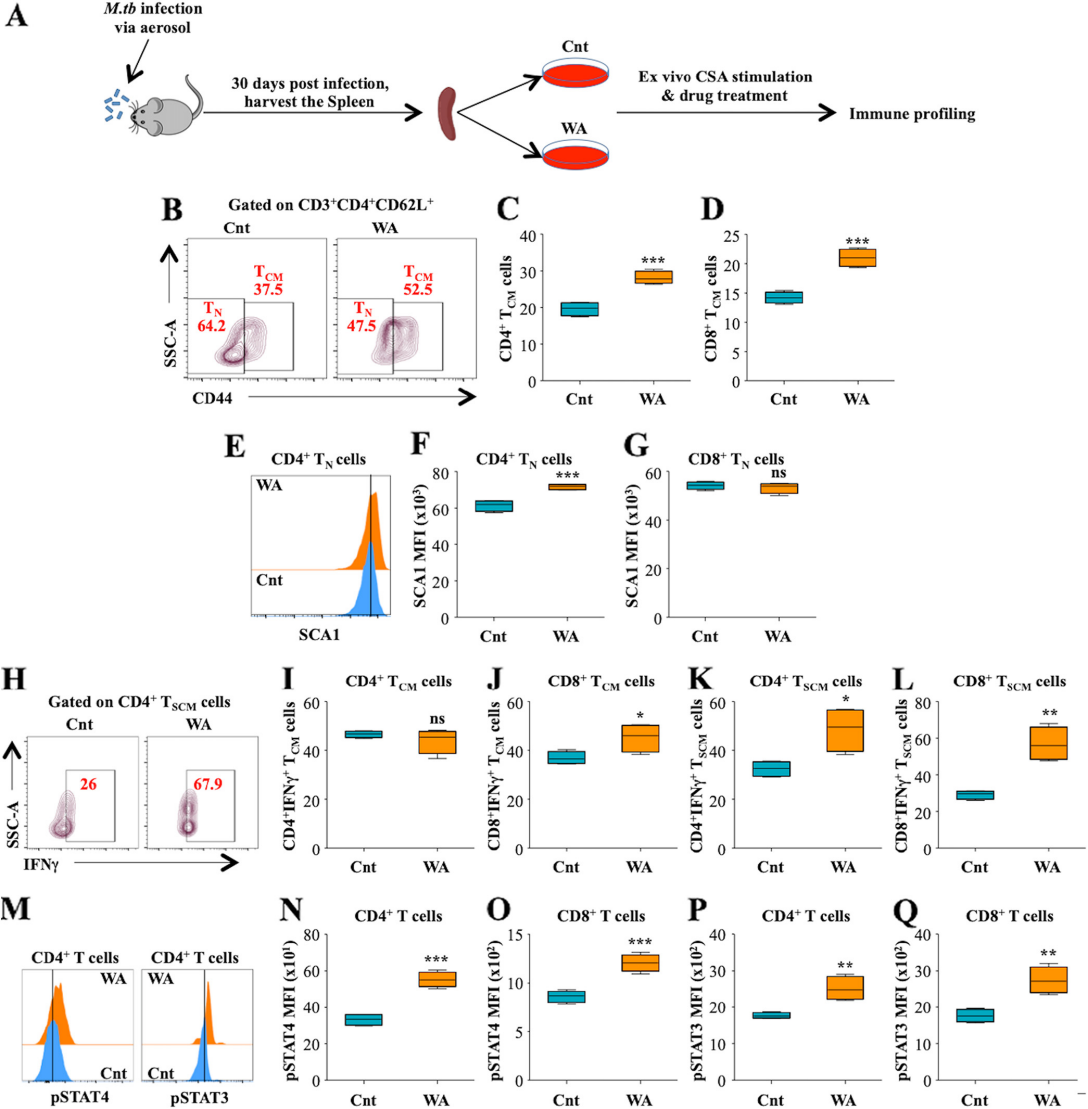
WA treatment modulates the STAT3 and STAT4 axis to enrich the memory responses against TB. (A) Schematic representation of the *ex vivo* experiment performed for immune profiling. Splenocytes isolated from M. tuberculosis-infected mice were stimulated with M. tuberculosis CSA and treated with WA (0.1 μg/mL) for 48 h. *Ex vivo*-stimulated splenocytes were surface stained with anti-CD3, -CD4, -CD8, -CD62L, -CD44, and -IFN-γ, followed by flow cytometry. (B) Contour representation of different T cell subsets. (C and D) Percentages of CD4^+^ T_CM_ cells (C) and CD8^+^ T_CM_ cells (D) with or without WA. (E) Histograms showing the expression of SCA1 in T_N_ cells with or without WA treatment. (F and G) Quantification of SCA1 MFI in CD4^+^ T_N_ cells (F) and CD8^+^ T_N_ cells (G) in control or WA-treated splenocytes. (H) Fluorescence-activated cell sorting (FACS) plot showing IFN-γ expression in CD4^+^ T_SCM_ cells. (I to L) Percentages of CD4^+^ IFN-γ^+^ T_CM_ cells (I), CD8^+^ IFN-γ^+^ T_CM_ cells (J), CD4^+^ IFN-γ^+^ T_SCM_ cells (K), and CD8^+^ IFN-γ^+^ T_SCM_ cells (L). *Ex vivo*-stimulated splenocytes were surface stained with anti-CD3, -CD4, and -CD8 and intracellular staining with pSTAT3 and pSTAT4. (M) Histograms showing the expression of pSTAT3 and pSTAT4 in CD4^+^ T cells with or without WA treatment. (N and O) pSTAT4 MFI in CD4^+^ T cells (N) and CD8^+^ T cells (O). (P and Q) pSTAT3 MFI in CD4^+^ T cells (P) and CD8^+^ T cells (Q). Data are representative of three independent experiments (*n* = 4).

To comprehend the molecular signaling associated with the enhancement of T cell memory responses after WA treatment, we isolated splenocytes from M. tuberculosis-infected mice and stimulated them with M. tuberculosis complete soluble antigen (CSA), followed by treatment with WA (0.1 μg/mL) for 48 h (Fig. 5A). In accordance with *in vivo* results, we found an increase in the percentages of CD4^+^ T_CM_ and CD8^+^ T_CM_ subsets (Fig. 5B to D). We further observed an augmented expression of SCA1^+^ (stem cell antigen-1) on the naive CD4^+^ subset after WA treatment (Fig. 5E to G). SCA1 is a marker of stem cell-like memory T (T_SCM_) cells, which have the ability to self-renew and the multipotent capacity to reconstitute the entire spectrum of memory and effector subsets (36). Furthermore, WA treatment enhanced the secretion of IFN-γ by T_CM_ and T_SCM_ memory subsets (Fig. 5H to L), which can provide an early inductive source of IFN-γ upon secondary infections for prompt bacterial clearance. Stimulation of T cell receptor (TCR) and induced signaling cascades such as the STAT3 and STAT4 pathways play a critical role in T cell differentiation and influencing memory responses against M. tuberculosis infection (37). STAT4 is indispensable for the induction of T_H_1 immune responses, while STAT3 is critical for the T_H_17 response against recurrent infections (38, 39) and establishment of T cell memory subsets (37). Many reports suggest that JAK/STAT signaling pathways play an important role in T cell memory responses (40, 41). STAT3 induces central memory T cells (42, 43), while STAT4 plays a critical role in the long-term T cell memory responses (44). Interestingly, WA treatment significantly enriched the percentages of pSTAT4- and pSTAT3-expressing CD4^+^ and CD8^+^ subsets (Fig. 5M to Q). The results hence imply that WA specifically stimulates memory T cell responses by activation of STAT3 and STAT4 signaling cascades.

### WA treatment lessens the extent of TB recurrence.

**FIG 6:**
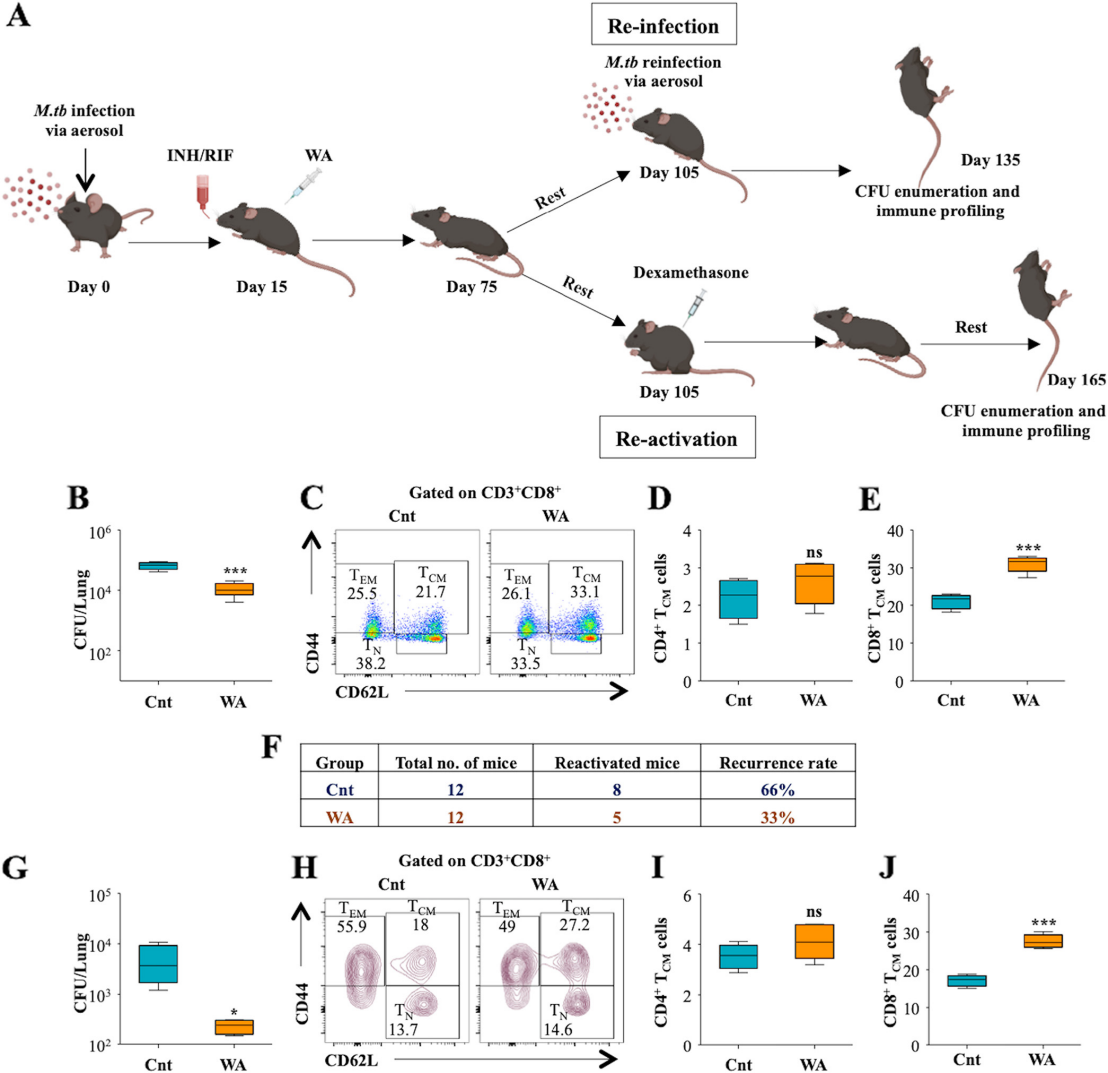
WA treatment alleviates the risk of TB reinfection and reactivation in the murine model of TB. (A) Schematic representation of reinfection and reactivation mouse model used in the study. (B) Bacterial burdens in the lungs of reinfected mouse groups. *Ex vivo*-stimulated single-cell suspensions of the lungs were stained with anti-CD3, -CD4, -CD8, -CD62L, and -CD44, followed by flow cytometry. (C) Dot plots representing T_CM_ and T_EM_ populations in the CD3^+^ CD8^+^ cells isolated from the lungs of reinfected mice. (D and E) Bar graphs showing the percentage of CD4^+^ T_CM_ (D) and CD8^+^ T_CM_ (E) cells in the lungs of reinfected mice. (F) Rate of disease relapse with and without WA treatment in the reactivation study. (G) Bacterial burdens in the lungs of relapsed animals. (H) Contour plots representing the memory T cell subsets (T_N_, T_CM_, and T_EM_) in CD3^+^ CD8^+^ cells in the spleens of the reactivation mouse groups. (I and J) Percentages of CD4^+^ T_CM_ (I) and CD8^+^ T_CM_ (J) cells in the spleen. Data are representative of two independent experiments. Values represent means ± SD (*n* = 4 to 6).

Memory T cells are vital for long-term protection against recurring infections, and lack of long-term immunity can cause TB relapse. Enriched pools of T_CM_ populations can efficiently drive prompt and robust antigen-specific immune responses to recurrent infections and prevent the possibility of reinfection. One of the major shortcomings of DOTS therapy is the heightened risk of disease reactivation upon immune dampening. Since our previous results demonstrated the generation of diverse memory T cell subsets upon WA treatment in M. tuberculosis-infected mice, we evaluated the impact of WA-instigated T memory populations against recurrent infection and reactivation of TB (Fig. 6A). To assess this, C57BL/6 mice were infected with H37Rv, followed by treatment with INH plus RIF (Cnt group) or INH plus RIF plus WA (WA group) for 60 days, following which the mice were put on rest for the next 30 days. For the reinfection experiment, the mice were rechallenged with H37Rv, and for reactivation, the mice were treated with immunosuppressive drug dexamethasone for 30 days, followed by enumeration of CFU and immune profiling. During reinfection, the mice which received INH plus RIF plus WA displayed a significant reduction in the bacterial loads in the lungs compared to the group which received INH plus RIF (Fig. 6B). Interestingly, immune profiling demonstrated enhancement in the percentage of CD8^+^ T_CM_ cells and no differences in CD4^+^ subsets in the lungs of WA-treated reinfected mice (Fig. 6C to E). In the case of reactivation, only 33% of mice (5 out of 12) in the WA group displayed disease relapse, compared to 66% mice (8 out of 12) in the control group (Fig. 6F). Moreover, even in the relapsed mice, the bacterial burdens in the lungs of WA-treated animals were significantly lower than in the control group (Fig. 6G). Further, WA-treated animals displayed an increased percentage of CD8^+^ T_CM_ cells, with no differences in CD4^+^ subsets in the spleen (Fig. 6H to J). These results lead us to conclude that WA treatment as an adjunct to DOTS offers diminution in relapse rates and augmented protection against recurrent M. tuberculosis infections by enriching the CD8^+^ T_CM_ population in the lungs of M. tuberculosis-infected mice.

## DISCUSSION

Upon M. tuberculosis infection, the host defenses instinctively restrain invasion by activating protective mechanisms to preserve the coherence of host physiology. However, absolute bacterial sterility is seldom accomplished and M. tuberculosis persists in the host in a dormant stage. In around 10% of the individuals, impairment of the defenses leads to colonization by M. tuberculosis and instigation of the life-threatening disease TB. Largely, we know that components of innate immunity prompt responses harmoniously with adaptive immune cell populations to constraint infection. Conversely, M. tuberculosis employs diverse stratagems to evade host-microbicidal forces (8). However, we have yet to uncover immune mechanisms that can drive long-term protection against M. tuberculosis infections, by maximizing pauci-inflammatory microbicidal responses for clearance of M. tuberculosis along with refrained tissue impairment. Progressive efforts are being made to establish efficacious immunomodulators, to reprogram host defenses for better clinical outcomes. This motivated us to retrospectively examine and harness the paramount knowledge of traditional medicine for progressive solutions to counteract the shortcomings associated with conventional ATT. The immunomodulatory potential of withaferin A (WA), the predominant bioactive constituent of the medicinal plant *Withania somnifera*, against numerous ailments has been characterized (27, 45). However, even with preliminary clinical data suggesting the prospects of *W. somnifera* for TB treatment (22), the efficacy and impact of WA against M. tuberculosis infection are yet to be established. Primarily, *ex vivo* analysis on murine peritoneal macrophages substantiated the antimycobacterial potential of WA against M. tuberculosis H37Rv. Upon inhalation, M. tuberculosis first encounters macrophages, which internalize the pathogen and instigate a cascade of immune signaling leading to activation of antigen-specific adaptive immune responses. Stimulation of antimycobacterial responses such as ROS generation, release of chemokines and cytokines to direct migration, and activation of immune cells by impelling signaling determine the progression of infection (8). WA treatment enhanced the activation of macrophages in response to M. tuberculosis infection, which was evident with augmented ROS levels, heightened levels of protective cytokines IL-1β, IL-6, and IL-23, instigation of p38 signaling, and increased costimulation. This was in line with cancer-related literature demonstrating robust growth-inhibitory effects of WA with induction of p38 signaling (46). Similarly, in TB, it has been delineated that the p38 signaling pathway contributes to macrophage activation-induced inflammation upon infection to prohibit intracellular survival of M. tuberculosis (29), which was evident in WA-treated infected murine macrophages. APCs provide protection against M. tuberculosis by internalizing and processing the bacteria and by activating T cell immunity. In our study, WA treatment increased the expression of activation markers on the surface of infected macrophages, leading to a prominent T cell activation and improved bacterial reduction under coculture conditions in which T cells were pretreated with WA followed by coculture with infected macrophages. Since conventional ATT dampens the host-protective immune response during the course of infection (4), use of an adjunct immunotherapeutic with ATT can overcome these shortcomings. In our study, we utilized WA with the front-line drug INH and observed improved pathogen clearance in infected macrophages treated with combination therapy. Furthermore, WA amended the potential of INH to activate infected macrophages for induction of protective cytokines in response to M. tuberculosis infection.

The therapeutic potential of WA in animal models has been evaluated in the past for diverse ailments, with no considerable adverse effects (28, 47), and previously established hepatoprotective effects of WA (48, 49) can be employed to overcome the shortcomings of TB treatment (34). Further validation of the antimycobacterial potential of WA was executed in the murine model against the virulent H37Rv laboratory strain of M. tuberculosis and drug-resistant clinical isolates. WA treatment alone and in conjunction with the frontline drug INH significantly led to a reduction in bacterial burden as well as amended pathological tissue damage induced by M. tuberculosis infection. WA treatment in mice has been shown to instigate cellular immune responses and T_H_1 polarization (23). In our study, we extensively assessed the immunological effect of WA treatment in the M. tuberculosis infection model. Consistent with accessible literature and our *ex vivo* results, WA treatment considerably enhanced the activation of innate immune cell populations (macrophages and dendritic cells). Furthermore, significant immunomodulation was apparent, with equivalent activation of adaptive immune cell populations. The dramatic increase in the percentage of activated CD4^+^ and CD8^+^ T lymphocytes upon cotreatment with INH further demonstrates the utility of WA as an adjunct to standard ATT.

In our comprehensive immunological analysis, we further evaluated the impact of WA treatment on the establishment of T memory cell populations against M. tuberculosis infection. Immunological memory is a strategic feature of the immune system to defend against repetitively encountered pathogens (50). It is well established that the memory T cell populations such as T_EM_ and T_CM_ play a critical role in the establishment of long-term protection against M. tuberculosis infections (51). Moreover, another memory T cell subset, T resident memory (T_RM_) cells, induce defensive responses at specific tissues, mainly prime sites of infection (52). T_RM_ cells perform immunoregulation by stimulation of IFN-γ and recruitment of memory T cells at the site of infection upon encountering pathogenic antigens (53). We demonstrate significant enrichment of T_CM_ and T_EM_ populations with a comparable reduction in the T_N_ population in WA-treated mice. Similarly, the percentage of the T_RM_ population was drastically enlarged in mice treated with WA alone or in combination with INH. While memory subsets contribute by instigating specific, heightened, and effective immune responses, cytokines play a vital role in the establishment and maintenance of immune memory subsets and orchestrating immune responses (54). IFN-γ plays a central role in the stimulation of host-protective immune responses in TB. Depletion of IFN-γ is strongly correlated with the dampening of defenses against M. tuberculosis in TB patients and animal models (55, 56). Analogously, cytokine IL-17 contributes vastly by inducing protection against recurrent infections by eliciting T_H_17 responses (57). Enrichment of the percentages of IFN-γ- and IL-17-secreting T cells in WA-treated mice further substantiates inclusive host-protective immunomodulation against M. tuberculosis. Inability to complete extended DOTS is linked with escalation in drug-resistant M. tuberculosis variants worldwide. Unmanageable DR-TB in patients with no therapeutic alternatives is a prevailing serious health threat in high-TB-burden nations (1, 3). WA treatment considerably reduced bacterial load, with consistent improvement in lung pathology, in MDR and XDR strain-infected mice. With no increase in the percentage of T cells, significantly heightened activation status of T cells was observed upon WA treatment. Furthermore, similar to the case with the drug-susceptible strain, augmented percentages of IFN-γ- and IL-17-secreting T cells were observed in WA-treated mice infected with drug-resistant M. tuberculosis strains. Furthermore, antimycobacterial immune responses induced upon WA treatment were confirmed to be M. tuberculosis-specific protective T cell responses by observing adoptively transferred protection and reduction in bacterial burden in infected Rag1^−/−^ mice.

To comprehend the mechanistic course of immune signaling by which WA treatment augmented memory T cell responses against M. tuberculosis infection, splenocytes harvested from M. tuberculosis-infected mice were cultured in the presence of WA. Consistent with the *in vivo* outcomes, WA treatment enhanced the percentages of CD4^+^ and CD8^+^ T_CM_ populations. Furthermore, an increase in IFN-γ-secreting CD8^+^ T_CM_ cells in response to WA treatment was also observed. Most importantly, WA treatment induced the expansion of the rare T stem cell memory population (T_SCM_), which can efficaciously expand and maintain the spectrum of T memory populations in response to infection (36). The establishment of T cell memory populations is reliant on antigenic stimulation of T cells and consequent intracellular signaling induced in response to infection (54). Diverse signaling pathways collectively determine the fate of immunological responses (58). Based on existing literature, we knew that WA can stimulate STAT signaling by instigating phosphorylation and activation of STAT3 (45). It is well established that STAT signaling can orchestrate the establishment and maintenance of memory cell populations in response to infections (43). It is known that STAT3 plays a vital role in the production and continuation of memory cell populations (43, 59) and that STAT4 is majorly involved at the site of infection and in stimulating tissue-resident memory responses (44). To evaluate the anticipated role of immunological signaling in the establishment of protective memory cell populations, we assessed the status of signal transducers in response to WA treatment. Augmentation of phosphorylated STAT-3 and STAT-4 in CD4^+^ and CD8^+^ T cells implied that the memory responses elicited by WA can be justified with induction of STAT-3 and STAT-4 signaling in response to M. tuberculosis infection.

**FIG 7:**
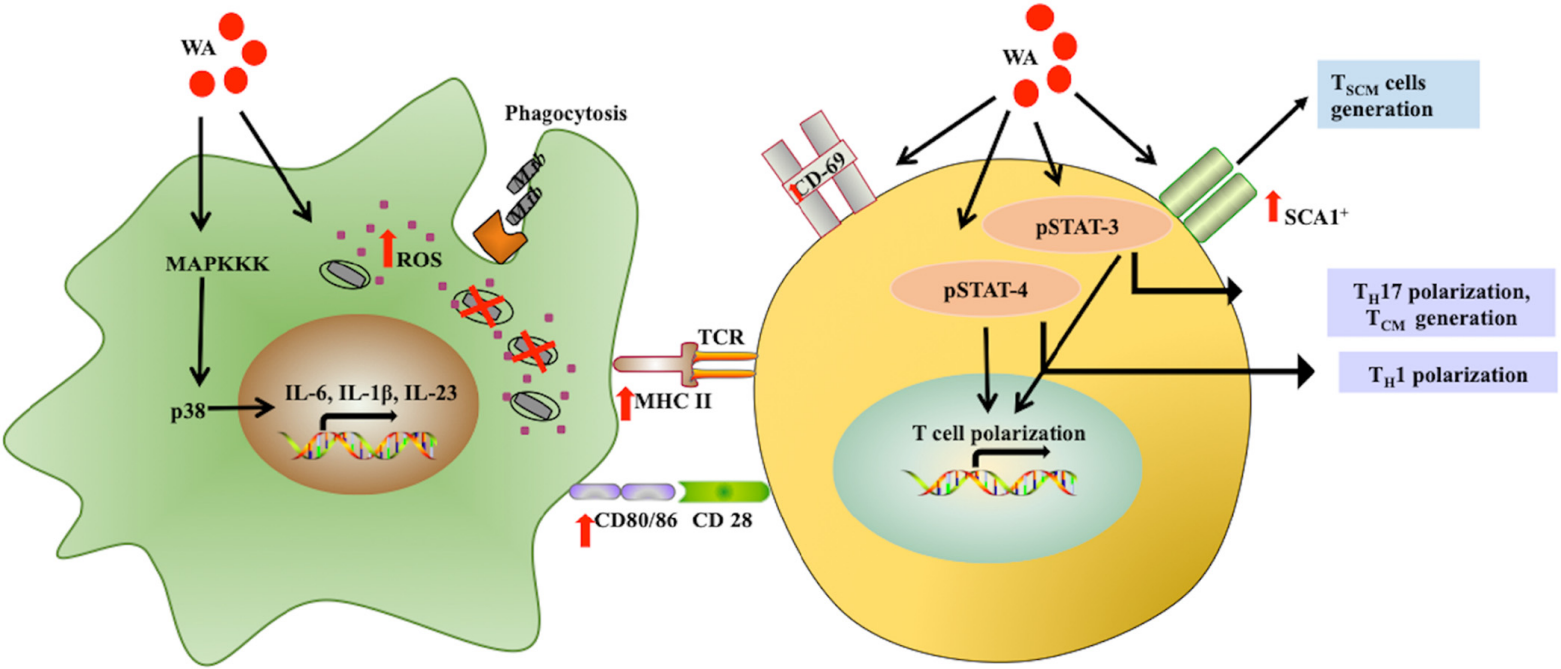
Proposed mechanism of WA-mediated impediment of M. tuberculosis survival. WA reprograms and activates host macrophages to an inflammatory M1 phenotype by upregulating ROS generation and the p38 MAPK signaling pathway. The enhanced ROS and upregulation of proinflammatory cytokine generation through the p38 MAPK pathway in the host macrophages culminate into a bactericidal milieu. The WA-mediated macrophage activation results in enhanced host T lymphocyte activation, as evidenced through upregulation of various activation markers. WA treatment further enhances host antimycobacterial adaptive immune responses through the polarization of T lymphocytes to proinflammatory T_H_1 and T_H_17 phenotypes. The treatment also upregulates various STAT signaling pathways in the T cells. This further enriches M. tuberculosis-specific memory T lymphocyte responses and generates long-term antimycobacterial protection with a decrease in TB recurrence as evidenced by the increase in T_SCM_ and T phenotype.

We further aimed to authenticate the enduring immunotherapeutic efficacy of WA in reinfection and reactivation mouse models of TB. In conjunction with the front-line ATT drugs INH and RIF, a significant reduction in bacterial load was observed in WA-treated mice with the expansion of CD8^+^ T_CM_ responses. Moreover, as a consequence of established continuing memory T cell populations, WA treatment drastically downgraded the recurrence rate, by 33%. Secondary immune responses are refined protective responses mounted by specific subpopulations which impart endurance to combat recurrent infections (3). Consequently, we ascertained the prospects of WA as an efficacious immunomodulatory phytochemical that can dramatically enhance lasting host-protective memory responses against M. tuberculosis infection (Fig. 7).

## MATERIALS AND METHODS

### Ethics statement.

Animal experiments were executed according to the guidelines approved by the Institutional Animal Ethics Committee of the International Centre for Genetic Engineering and Biotechnology (ICGEB; New Delhi, India) (approval ICGEB/IAEC/18092021/IMB-19) and the Department of Biotechnology (DBT), Government of India. All mice used for the experiments were ethically sacrificed by asphyxiation with carbon dioxide according to the institutional and DBT regulations.

### Mice.

C57BL/6 mice and Rag1^−/−^ mice (6 to 8 weeks; body weight, 20 to 25 g) were maintained at a pathogen-free animal facility at ICGEB, New Delhi, India. Mice used for infections were acquired as per the experimental requirements.

### Bacteria.

Mid-log-phase cultures of M. tuberculosis strains (H37Rv, Rv-GFP, JAL-2261 [MDR], and MYC-431 [XDR]) were used in the study. Mycobacterial strains were grown in 7H9 (Middlebrook; Difco, USA) medium supplemented with 10% oleic acid, albumin, dextrose, and catalase (OADC; Difco), 0.05% Tween 80, and 0.2% glycerol. Bacterial stocks used for infection experiments were prepared in 20% glycerol and were cryopreserved at −80°C.

### alamarBlue assay.

A mid-log-phase bacterial culture (optical density at 600 nm [OD_600_], 0.6 to 0.8) was grown and added at a final OD_600_ of 0.01 to a 96-well plate containing 2-fold serially diluted drug followed by incubation for 5 days at 37°C. INH was used as a positive control. alamarBlue dye (1×; 10 μL/well; Thermo Fisher Scientific) was added and a color change from blue to pink was visualized after 16 h of incubation. Wells containing only medium and no bacilli were treated as the negative-control group (blue), and wells with only bacilli without any drug were treated as the positive-control group (pink).

### Isolation of mouse peritoneal macrophages.

A 1.5-mL volume of 4% thioglycolate broth was injected intraperitoneally into 6- to 8-week-old C57BL/6 mice. On the 5th day postinjection, macrophages were isolated from peritoneal lavage fluid and washed with chilled phosphate-buffered saline (PBS). Cells were stained with trypan blue and were counted using a hemocytometer chamber. Macrophages were further suspended in RPMI 1640 medium supplemented with 10% heat-inactivated fetal calf serum and 1% antibiotic (penicillin-streptomycin [pen-strep]). Cells were seeded at a density of 1 × 10^6^ cells/mL. After overnight incubation at 37°C and 5% CO_2_, nonadherent cells were washed off using 1× sterile PBS and adherent cells were infected with M. tuberculosis strains at an MOI of 1:1.

### *Ex vivo* infection and CellROX assay.

Bacterial cryostocks were revived and a single-cell suspension was made to infect peritoneal macrophages at an MOI of 1:1. At 4 h postinfection, cells were washed twice with sterile 1× PBS to terminate M. tuberculosis infection. Murine peritoneal macrophages were then treated with 0.5 μg/mL of WA and were incubated at 37°C for different times for CFU enumeration (60), immunoblotting, RNA isolation, or flow cytometry. To determine the intracellular ROS, CellROX dye (Thermo Fisher Scientific) was used as per the protocol supplied by manufacturer.

### PKH26 staining.

A single-cell suspension was made from axenic mid-log-phase bacterial cultures of drug-resistant M. tuberculosis strains JAL-2261 (MDR) and MYC-431 (XDR) in 1× PBS. Bacilli were stained with the PKH26 red fluorescent cell linker kit (Sigma-Aldrich) as per the protocol provided by the manufacturer. Peritoneal macrophages were infected with stained drug-resistant strains at an MOI of 1:1 separately. Infection was terminated after 4 h, and peritoneal macrophages were treated with 0.5 μg/mL WA for 48 h. To determine the percent infection, cells were analyzed via flow cytometry.

### Mouse infection and CFU estimation.

For *in vivo* experimentations, mice were infected with H37Rv and the MDR and XDR strains of M. tuberculosis by the aerosol route employing a Madison aerosol chamber (University of Wisconsin, Madison, WI) with the nebulizer precalibrated to transmit 110 bacilli to the lungs of each mouse. Mycobacterial stocks recovered from −80°C were quickly thawed and a 15-mL single-cell suspension was prepared using a 26-gauge needle. Five or six mice from each group were sacrificed at different time points, and organs were homogenized in sterile PBS. Homogenates of lung and spleen at different dilutions were plated onto 7H11 plates (Middlebrook; Difco, USA) containing 10% OADC (Difco) and incubated at 37°C. After 21 to 28 days, the colonies were counted.

### Drug administration.

For *ex vivo* experiments, peritoneal macrophages were treated with 0.5 μg/mL of WA (Sigma-Aldrich). For mouse studies, 2 mg/kg of WA in 100 μL of PBS was administered intraperitoneally every alternate day during the 60 days of treatment. WA dosage was decided based on the previous literature (61, 62). INH (0.1 g/L) and/or RIF (0.06 g/L) was constantly given in the drinking water, which was changed every alternative day.

### T cell adoptive transfer.

To perform adoptive-transfer experiments, a single-cell suspension using autoclaved frosted slides was prepared from isolated lungs of M. tuberculosis-infected and treated mice in complete RPMI 1640 medium. Poststaining, the CD3^+^ CD4^+^T cells were sorted by FACS Aria (BD Biosciences) and cultured overnight. Around one million CD4^+^ cells were injected into naive Rag1^−/−^ mice, which were infected with a low-dose aerosol of M. tuberculosis after 5 days. The mice were then euthanized to determine the bacterial survival in the lungs and the spleen.

### Flow cytometry.

To obtain a single-cell suspension, lung and spleen samples derived from different groups of mice were macerated using frosted slides in ice-cold RPMI 1640 medium supplemented with 10% fetal bovine serum (FBS). Further to remove red blood cells (RBCs), lysis buffer was used and cells were washed with 10% RPMI 1640 medium. For surface staining, cells were stimulated with 10 μg/mL of M. tuberculosis complete soluble antigen (CSA). For intracellular cytokine staining, the cells were treated with 0.5 μg/mL of brefeldin A and 0.5 μg/mL of monensin for 4 h prior to cell extraction. Then the cells were washed twice with FACS buffer (PBS plus 3% FBS), stained with surface antibodies, and fixed with 100 μL of fixation buffer (Biolegend) for 30 min. Intracellular staining was performed using 1× permeabilization buffer (Biolegend) and staining with fluorescently labeled anti-cytokine antibodies. Tagged secondary antibody with Alexa Fluor 488 was used for unlabeled primary antibodies. The samples were then analyzed by flow cytometry (BD LSRFortessa cell analyzer; BD Biosciences) followed by data analysis via FlowJo (Tree Star, USA).

### Antibodies.

The following anti-mouse antibodies were used: CD3-Pacific Blue, CD4-peridinin chlorophyll protein (PerCP)-Cy5.5, CD8-allophycocyanin (APC)-Cy7, CD69-phycoerythrin (PE), CD44-fluorescein isothiocyanate (FITC), CD62L-APC, CD103-PE-Cy7, CD69-FITC, IFN-γ-APC, IFN-γ-BV510, IL-17-PE-Cy7, IL-17-BV650, CD11b-APC-Cy7, CD11c-APC, CD80-FITC, CD86-PerCP-Cy5.5, CD40-PE, CD4-PE, CD4-APC, CD4-FITC, CD3-BV510, and CD3-BV650 from Biolegend, USA.

Alexa Fluor 647 secondary antibody or isotype control IgG-APC was from Abcam.

p-P38, P38, pSTAT3, pSTAT4, and β-tubulin were obtained from Cell Signaling Technology.

I-A(b)_4-17_ESAT-6 MHC-II tetramer to analyze the M. tuberculosis-specific CD4^+^ T cell responses was procured from NIH Tetramer Facility, USA (USA).

### Histopathology.

Lungs of infected animals were harvested at different time points, fixed in 10% buffered formalin solution, and further embedded on 5-μm-thick paraffin wax. Hematoxylin and eosin (H&E) staining was done on 5-μm-thick paraffin-embedded tissues. Five fields were screened to deduce the granulomas for each mouse in all groups. Images are representative of all the observed sections.

### Reinfection and reactivation experiments.

To evaluate the reinfection and reactivation susceptibility, C57BL/6 mice were infected with a low-dose aerosol of M. tuberculosis strain H37Rv. At 15 days postinfection, mice were administered INH (0.1 g/L) and/or RIF (0.06 g/L) in drinking water, either alone or with 2 mg/kg of WA, which was given intraperitoneally every alternative day for 60 days. The mice from different groups were then rested for 30 days. For reinfection studies, mice were again challenged with a low dose of M. tuberculosis and after 30 days of secondary infection, the mice were sacrificed to evaluate the CFU and immunological profiles. In reactivation model, mice were treated with dexamethasone (5 mg/kg administered intraperitoneally) three times per week for 30 days, after which the mice from each group were euthanized for CFU estimation and immune profiling.

### Hepatotoxicity assays.

To assess the drug-induced hepatotoxicity, serum was collected from randomly chosen mice from each group. Assay to examine hepatotoxicity associated with therapy was performed using diagnostic kits obtained from Span Diagnostic Limited (India), in accordance with the manufacturer’s protocol.

### Immunoblotting.

For protein isolation from peritoneal macrophages, first radioimmunoprecipitation assay (RIPA) lysis buffer (50 mM Tris [pH 8.0], 150 mM NaCl, 1.0% NP-40, 0.5% sodium deoxycholate, 0.1% SDS) containing 1× protease inhibitor cocktail was prepared for whole-cell lysates. Samples were electrophoresed on 10% polyacrylamide gels (SDS-PAGE) and electroblotted onto a polyvinylidene difluoride (PVDF) membrane. Further blocking was done using 5% bovine serum albumin (BSA) solution in PBS with 0.1% Tween 20 (PBST), followed by overnight probing at 4°C with the respective antibodies. Blots were developed using chemiluminescent horseradish peroxidase (HRP) substrate (ECL, Millipore) and visualized on an ImageQuant LAS 500 instrument.

### qPCR evaluation.

Whole RNA from macrophages was extracted by following the standard RNA isolation protocol (63). Further cDNA synthesis was done with an iScript cDNA synthesis kit. Real-time quantitative PCR (qPCR) was performed using SYBR green master mix (Bio-Rad) and a Bio-Rad real-time thermal cycler (Bio-Rad, USA). The reaction was set up according to the manufacturer’s protocol. The primer sequences used in the study are given in Table 1.

**TABLE 1:** Primers used in this study

Primer	Sequence (5′–3′)
IL-6 forward primer	CCGGAGAGGAGACTTCACAG
IL-6 reverse primer	TCCACGATTTCCCAGAGAAC
IL-1β forward primer	CCCAAGCAATACCCAAAGAA
IL-1β reverse primer	GCTTGTGCTCTGCTTGTGAG
IL-23 forward primer	AATAATGTGCCCCGTATCCA
IL-23 reverse primer	AGGCTCCCCTTTGAAGATGT
IL-12p40 forward primer	AAGGAACAGTGGGTGTCCAG
IL-12p40 reverse primer	GGAGACACCAGCAAAACGAT
TNF-α forward primer	TAGCCAGGAGGGAGAACAGA
TNF-α reverse primer	TTTTCTGGAGGGAGATGTGG
GAPDH^*a*^ forward primer	AACTTTGGCATTGTGGAAGG
GAPDH reverse primer	GGATGCAGGGATGATGTTCT

a GAPDH, glyceraldehyde-3-phosphate dehydrogenase.

### Statistical analysis.

GraphPad Prism software was utilized for the analysis of all the experimental results. Significant outcomes among the groups were determined by 2-tailed unpaired Student’s *t* test or one-way analysis of variance (ANOVA). In figures, significant differences are indicated as follows: *, *P* < 0.05; **, *P* < 0.005; and ***, *P* < 0.0005.
