# Supplementary material for: Withaferin A Protects against Primary and Recurrent Tuberculosis by Modulating Mycobacterium-Specific Host Immune Responses
Source: Microbiol Spectr. 2023 Mar 14;11(2):e00583-23. doi: 10.1128/spectrum.00583-23 (PMC10100980; doi:10.1128/spectrum.00583-23)
Supplement: Supplemental file 1 — Supplemental material. Download spectrum.00583-23-s0001.pdf, PDF file, 3.7 MB [file spectrum.00583-23-s0001.pdf]

SUPPLEMENTARY FIGURE LEGENDS

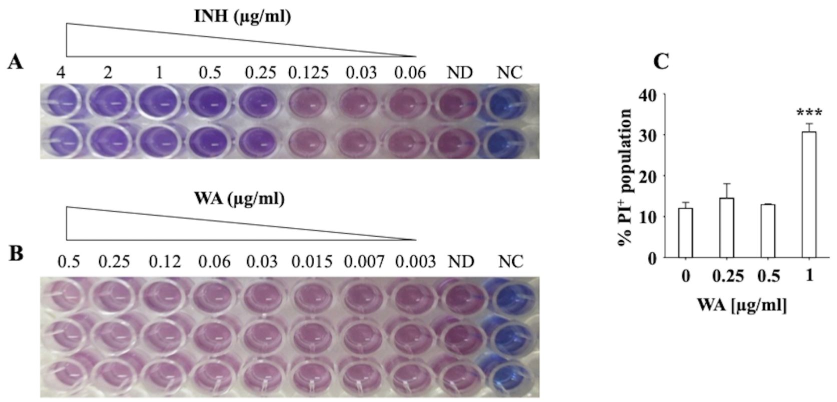

Supplementary Figure 1: WA does not exhibit direct anti-mycobacterial activity. Alamar Blue assay to assess the anti-mycobacterial activity of **(A)** INH and **(B)** WA. **(C)** Mouse peritoneal macrophages were treated with different concentrations of WA followed by Propidium iodide staining to evaluate the cytotoxic effect of WA. The data shown is representative of three independent experiments. * $p<0.05$, ** $p<0.005$, *** $p<0.0005$.

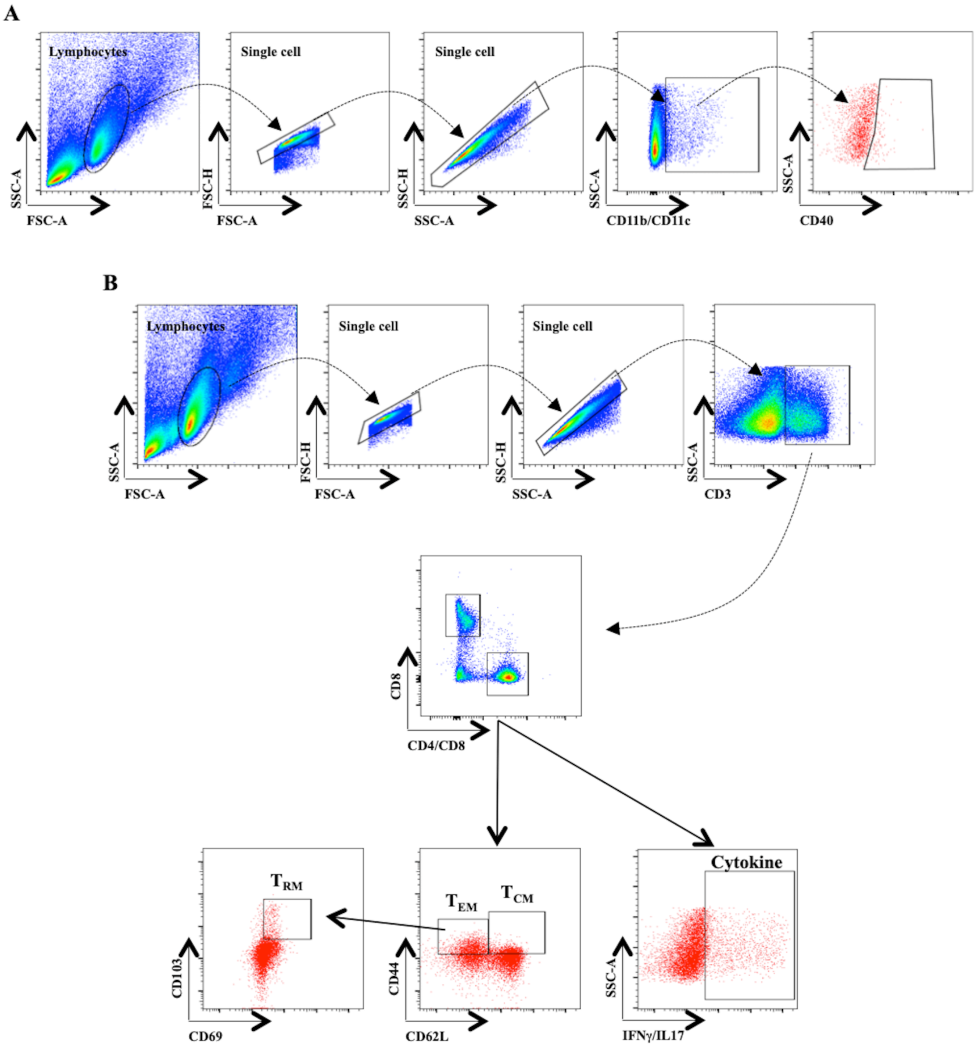

Supplementary Figure 2: Gating strategies to show the multi-color flow cytometry performed on myeloid and lymphoid cells. Cells were isolated from the lungs and the spleen of infected mice and stained with fluorescence antibodies tagged with monocytic and lymphocytic markers (see Methods). **(A)** Flow cytometry staining to show the CD40 co-stimulatory molecules on CD11b^{+} macrophages. **(B)** Gating strategy employed to characterize the T cells from the lungs and spleen of infected animals.

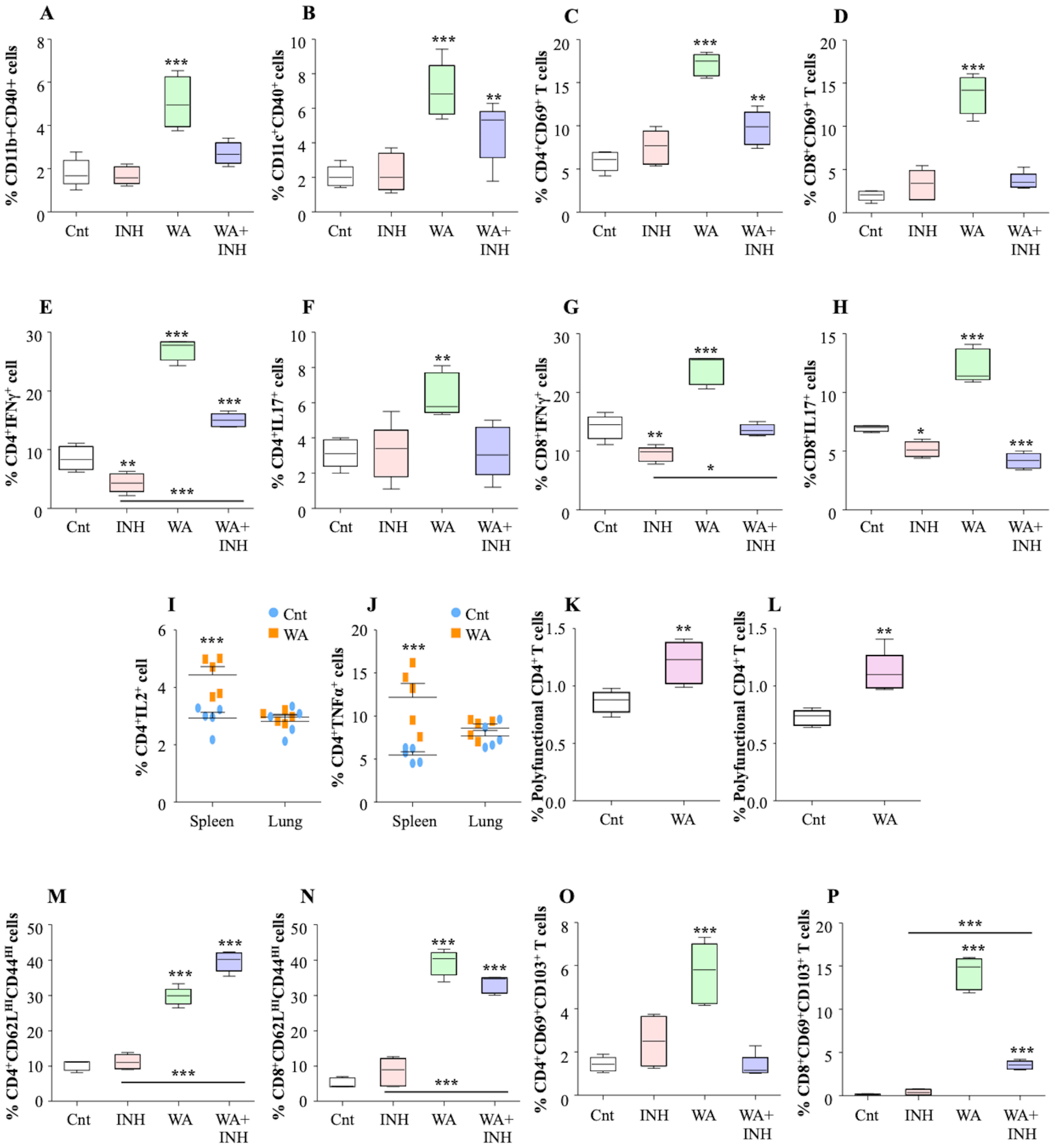

Supplementary Figure 3: WA treatment enriches the *M.tb* specific immune responses in the spleen of the *M.tb* infected mice. Costimulatory molecule CD40 expression on **(A)** the macrophages ($CD11b^+$) and **(B)** the dendritic cells ($CD11c^+$) in the spleen of infected animals. Percentage activation (CD69 expression) on **(C)** the $CD4^+$ and **(D)** the $CD8^+$ T cells in the infected spleen mice. **(E-H)** *Ex vivo* stimulated splenocytes were stained with anti-CD3, -CD4, -CD8, -IFNγ, and -IL17 and analyzed by flow cytometry. Percentage of **(E)** IFNγ producing $CD4^+$ T cells and **(F)** IL17 producing $CD4^+$ T cells in the spleen of infected mice. Percentage of $CD8^+$

T cells expressing **(G)** IFNγ and **(H)** IL17 in the spleen of infected mice. **(I-L)** *Ex vivo* stimulated lung and spleen cells isolated from infected and WA treated mice were stained with anti-CD3, -CD4, -CD8, -IFNγ, -IL17, -IL2 and -TNFα and analyzed by flow cytometry. Percentage of **(I)** IL2 producing $CD4^+$ T cells and **(J)** TNFα producing $CD4^+$ T cells in the spleen and the lungs of infected mice. Percentage of polyfunctional $CD4^+$ T cells expressing IFNγ, IL17, IL2 and TNFα in **(K)** the spleen and **(L)** the lungs of infected animals. **(M-P)** T lymphocytes isolated from the spleen of the infected mice were surface-stained with anti-CD3, -CD4, -CD8, -CD44, -CD62L, -CD69, and -CD103 antibodies for central memory and resident memory T cell responses. Percentage of **(M)** $CD4^+$ T_{CM} cells, **(N)** $CD8^+$ T_{CM} cells, **(O)** $CD4^+$ T_{RM} cells and **(P)** $CD8^+$ T_{RM} cells. Cnt: Control. WA: Withaferin A treated. INH: Isoniazid treated. WA+INH: Withaferin A and Isoniazid treated. The data shown is representative of two independent experiments with five mice per group. * $p<0.05$, ** $p<0.005$, *** $p<0.0005$.

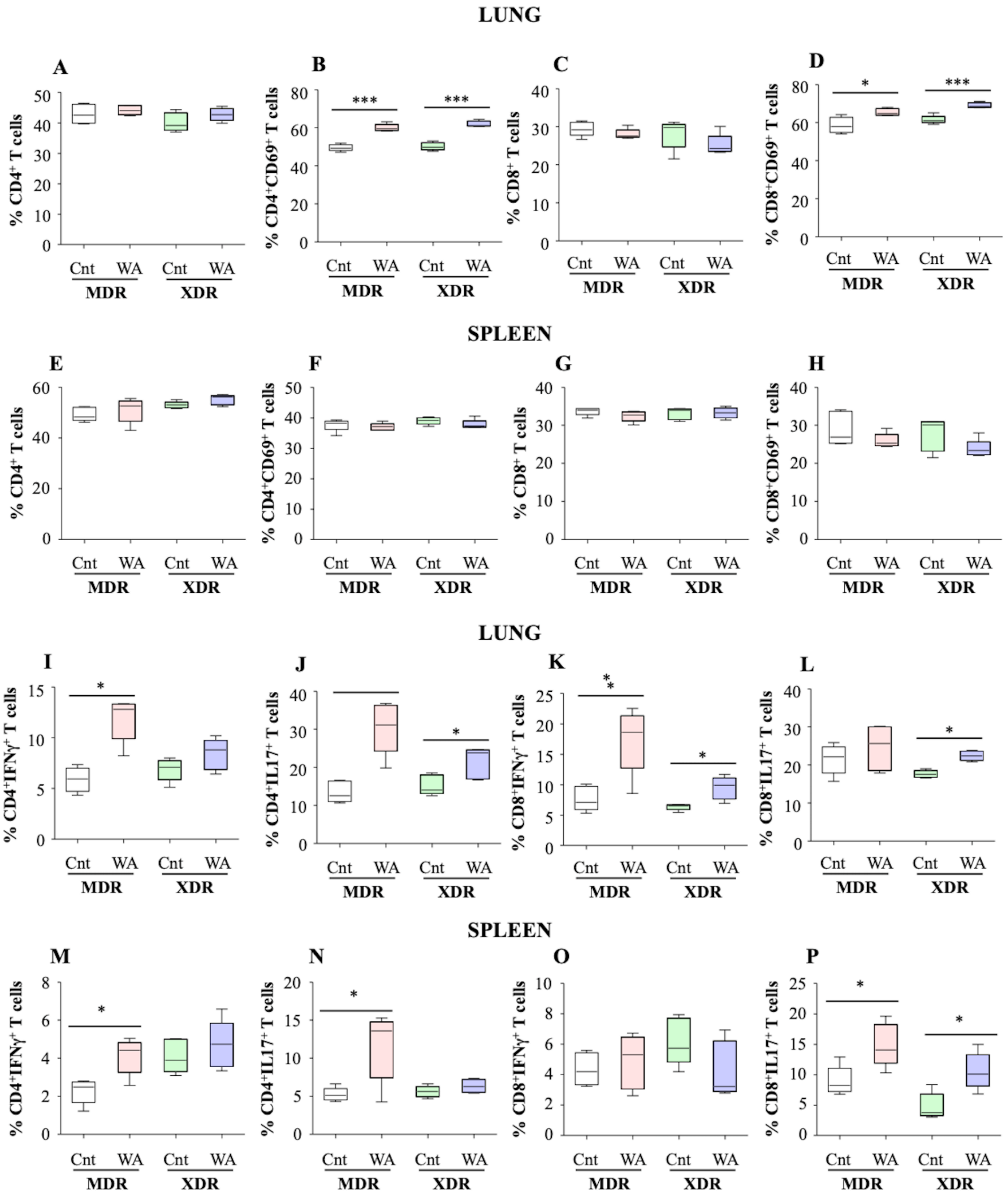

Supplementary Figure 4: WA treatment induces protective immune responses against MDR and XDR TB. Cells isolated from the lungs and spleen of the MDR and XDR infected mice were stained with anti-CD3, -CD4, -CD8, -CD69, -IFNγ and -IL17 antibodies followed by flow cytometry. Percentage of **(A)** $CD4^+$ T cells, **(B)** $CD4^+CD69^+$ T cells, **(C)** $CD8^+$ T cells, **(D)** $CD8^+CD69^+$ T cells, in the lungs of MDR infected mice. Percentage of **(E)** $CD4^+$ T cells, **(F)** $CD4^+CD69^+$ T cells, **(G)** $CD8^+$ T cells, **(H)** $CD8^+CD69^+$ T cells, in the spleen of MDR infected mice. Percentage of **(I)** $CD4^+IFN\gamma^+$ T cells, **(J)** $CD4^+IL17^+$ T cells, **(K)** $CD8^+IFN\gamma^+$ T cells and

(L) $CD8^+IL17^+$ T cells in the lung of XDR infected animals. Percentage of **(M)** $CD4^+IFN\gamma^+$ T cells, **(N)** $CD4^+IL17^+$ T cells, **(O)** $CD8^+IFN\gamma^+$ T cells and **(P)** $CD8^+IL17^+$ T cells in the spleen of XDR infected animals. Cnt: Control. WA: Withaferin A treated. The experiment was performed at least two times. The data shown represents mean ± SD (n=5). * $p<0.05$, ** $p<0.005$, *** $p<0.0005$.

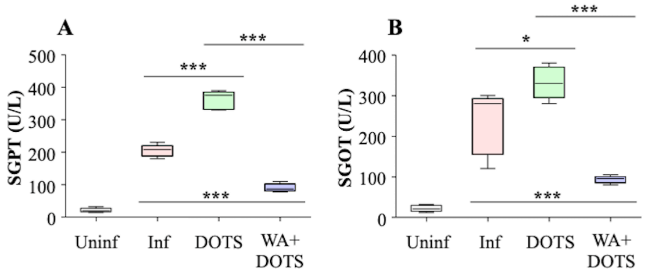

Supplementary Figure 5: WA treatment alleviates DOTS associated hepatotoxicity. Serum isolated from randomly chosen mice of different study groups 60 days post-treatment were processed for estimation of **(A)** serum SGPT activity and **(B)** serum SGOT activity. The data shown represents mean ± SD (n=5). * $p<0.05$, ** $p<0.005$, *** $p<0.0005$.
